# Extraction of Pectin from Satsuma Mandarin Peel: A Comparison of High Hydrostatic Pressure and Conventional Extractions in Different Acids

**DOI:** 10.3390/molecules27123747

**Published:** 2022-06-10

**Authors:** Xingke Duan, Yu Zhu, Congying Shu, Jihui Gao, Fengxia Liu, Siyi Pan

**Affiliations:** 1 College of Food Science and Technology, Huazhong Agricultural University, Wuhan 430070, China; a13223034502@163.com (X.D.); a951478637@foxmail.com (Y.Z.); xia870529@163.com (C.S.); hu4596252@163.com (J.G.); pansiyi@mail.hzau.edu.cn (S.P.); 2 Key Laboratory of Environment Correlative Dietology, Ministry of Education, Wuhan 430070, China; 3 Hubei Key Laboratory of Fruit & Vegetable Processing & Quality Control, Huazhong Agricultural University, Wuhan 430070, China

**Keywords:** Satsuma mandarin, pectin, high hydrostatic pressure, citric acid, structural properties, emulsifying properties

## Abstract

Satsuma mandarin peel pectin was extracted by high hydrostatic pressure-assisted citric acid (HHPCP) or hydrochloric acid (HHPHP), and the physiochemical, structural, rheological and emulsifying characteristics were compared to those from conventional citric acid (CCP) and hydrochloric acid (CHP). Results showed that HHP and citric acid could both increase the pectin yield, and HHPCP had the highest yield (18.99%). Structural characterization, including NMR and FTIR, demonstrated that HHPHP showed higher *Mw* than the other pectins. The viscosity of the pectin treated with HHP was higher than that obtained with the conventional method, with HHPHP exhibiting significantly higher viscosity. Interestingly, all the pectin emulsions showed small particle mean diameters (D_4,3_ being 0.2–1.3 μm) and extremely good emulsifying stability with centrifugation and 30-day storage assays, all being 100%. Satsuma mandarin peel could become a highly promising pectin source with good emulsifying properties, and HHP-assisted acid could be a more efficient method for pectin extraction.

## 1. Introduction

Pectin is a heterogeneous complex polysaccharide found in the primary cell wall and middle lamella of most plants, especially higher plants [1]. It is generally composed of D-galacturonic acid with α-1,4glycosidic bonds, which determine its functional properties. Their structure and functions are mainly depended on the sources [2,3] and extraction conditions [4].

At present, citrus peel [5] and apple pomace [1] are the main sources of commercial pectin. China has become a leading country for citrus production [6]. Mandarin (*Citrus reticulata*), the wide-skinned citrus variety, is ranked first in total citrus production in China (67%), with Satsuma mandarin (*Citrus unshiu*) being the main cultivar. It is one of the good varieties developed and promoted in China, and at present, Satsuma mandarin has become the main planting species in China and Japan [7]. With the vigorous development of China’s citrus industry, the high-value utilization of citrus peel has gradually become a vital problem. Except for a small amount of dried tangerine peel processing, most of the citrus peels are directly discarded and buried, which will cause serious environmental problems due to microbial corruption and carbon dioxide emissions [8]. Numerous studies have shown that citrus peels such as orange peel [9], grapefruit peel [10], lime peel [11] etc. are promising sources of pectin. However, there has been little research on the physicochemical and functional properties of pectin from Satsuma mandarin peels.

Besides, traditional pectin extraction is usually carried out in acidic conditions using mineral acids [12] or organic acids [12,13] as extraction solvents. Mineral acid extraction has the characteristics of high acidity and strong corrosion, which may cause degradation of pectin and environment pollution [11]. Furthermore, the use of mineral acids may introduce toxic elements into the final products [14]. In recent years, scholars have reported that organic acids with chelating properties had the advantages of mild extraction and less degradation of pectin. For example, it was reported that the yields and degree of esterification (DE) of pectin from citric acid were higher than those from hydrochloric acid and sulfuric acid [12,13]. Moreover, many scholars have explored novel extraction methods to obtain pectin with higher yield and better functional properties. Compared to heating in the extraction process, methods based on high hydrostatic pressure (HHP) processing are generally more environmentally friendly, more efficient, and more economical. HHP, as an important non-thermal technology, has been used to inactivate microorganisms in fruits, juices and vegetables [15]. Usually, HHP does not change the covalent bonds of food components but only affects noncovalent bonds such as hydrogen bonds, van der Waals forces and hydrophobicity [16]. It has been reported that HHP generally did not damage the main chain of pectin and can increase the viscosity of pectin solutions [17]. HHP treatment could result in significantly higher yields of lime peel pectin than aqueous extraction [11]. Xie et al. [18] found that the DE and *Mw* of pectin were decreased, accompanied by increased viscosity and improved emulsifying properties after HHP treatment. Peng et al. [17] found that the weight-average molar mass of sugar beet pectin decreased during HHP processing (≥250 MPa), and that the DE decreased if the system pH > 7. Nonetheless, previous studies mainly focused on HHP alone or assisted with mineral acid for pectin extraction or modification, and the combined effect of HHP and organic acids on extraction and characteristics of pectin was less studied.

Thus, the aims of this paper were to: (I) characterize pectin sourced from Satsuma mandarin peels, with regards to physiochemical, rheological and emulsifying properties; (II) comparatively investigate the effects of HHP on pectin extraction, in different solvents (citric acid and hydrochloric acid). This study may provide a theoretical basis for the high-value comprehensive utilization of by-products of Satsuma mandarin and expand the application of HHP in the efficient extraction of pectin.

## 2. Experimental Section

### 2.1. Materials

Fresh Satsuma mandarin fruit was purchased from the local market (Wuhan, China). D-galacturonic acid and phenylphenol were obtained from Sigma-Aldrich (St. Louis, MO, USA). All other chemicals in this study were of analytical grade and purchased from Sinopharm Chemical Reagent Co., Ltd. (Shanghai, China).

### 2.2. Preparation of the Peel Powder Sample

Satsuma mandarin fruit was manually peeled, and the peels were steamed at 100 °C for 5 min to destroy endogenous enzymes and then freeze-dried with a vacuum freeze dryer. After that, the dried sample was pulverized using an electric grinder (Zhejiang Industry and Trade Co., Ltd., Hangzhou, China) and then passed through a 40-mesh sieve. The peel powder was vacuum-packed and placed in a desiccator for use.

### 2.3. Pectin Extraction and Purification

#### 2.3.1. High Hydrostatic Pressure Extraction (HHPE)

HHPE was carried out based on the previous approach described by Guo et al. [19] with some modifications, using a hydrostatic pressure vessel (Baotou Kefa High Pressure Technology Co., Ltd., Baotou, China). The peel powders were dissolved in deionized water at the ratio of 1:50, and then, pH was adjusted to 1.4 by citric acid (or hydrochloric acid). Thereafter, the mixture was treated in the HHP vessel (500 MPa, 10 min). After that, the extraction solution was centrifuged at 8000× *g* for 10 min. The supernatant was collected and mixed with a third volume of absolute ethanol overnight. The wet pectin was filtered through 400-mesh gauze and washed three times with 30 mL absolute ethanol, acetone, and absolute ethanol, respectively, to remove the pigment, monosaccharides and disaccharides. Then, the pectin was obtained by freeze-drying, weighed and calculated. The pectin prepared using HHP extraction with citric acid (or hydrochloric acid) was recorded as HHPCP (or HHPHP).

#### 2.3.2. Conventional Extraction (CE)

The method of CE was carried out based on the previous approach of Wang, Huang, Fan, Zhao, Hu, Xu, Pan and Liu [4] with slight modifications. The peel powders were dissolved in deionized water at the ratio of 1:50, and then pH was adjusted to 1.4 by citric acid (or hydrochloric acid), respectively. Thereafter, the mixture was placed in a constant temperature water bath at 85 °C for 70 min and then cooled to room temperature. The following process was the same as those described in Section 2.3.1. The pectin prepared using conventional extraction with citric acid (or hydrochloric acid) was recorded as CCP (or CHP). The extraction yield (Y) was calculated according to the following formula:

Y (%, *w*/*w*) = *W*_1_/*W*_0_ × 100%

where *W*_1_ was the pectin weight (g), and *W*_0_ was the weight of Satsuma mandarin peel powder used (g).

### 2.4. Physicochemical Properties of Different Pectins

The GalA content of pectin was determined by the colorimetric method as described by Blumenkrantz and Asboe-Hansen [20], using a spectrophotometer at 520 nm (UV-762, Lingguang, Shanghai, China). Results were expressed as D-galacturonic acid equivalents in percent. The DE values of pectin were measured based on the titrimetric method using NaOH according to the Food Chemical Codex (FCC, 2004) [17], with slight medications. The end point of the titration with NaOH was changed to pH = 8.1. The *Mw* values and molecular weight distribution (MWD) of pectins were evaluated using high performance size exclusion chromatography with a refractive index detector (HPSECRID, Agilent 1100, Agilent Technologies, Palo Alto, CA, USA) as described by Wang, Huang, Fan, Zhao, Hu, Xu, Pan and Liu [4]. A Shodex OHpak SB-804 HQ column was used with a Shodex OHpak SB-G guard column. The eluent was 0.05 M NaNO_3_ solution, which containing 0.5 g/L NaN_3_. The flow rate was set to 0.5 mL/min, and the column temperature was set to 35 °C. Pullulan standards with different *Mw* of 0.186, 10, 40, 70, 500, and 2000 kDa (Showa Denko KK Inc., Kawasaki, Japan) were selected as the calibration standards.

### 2.5. Fourier-Transform Infrared (FTIR) Spectroscopy

FTIR spectroscopy measurements were performed using a Fourier Transform Infrared Spectrometer (Thermo Fisher Scientific, Madison, MA, USA), according to the method of Xie et al. [18]. Approximately 2 mg of pectin sample was taken and ground to a fine powder, mixed with KBr at a ratio of 1:100. Then, the mixture was placed in a dry agate mill, ground and compressed. After removal, a transparent sample sheet was obtained, and the infrared spectrum was scanned by a Fourier-transform infrared spectrometer with the scanning range from 500 to 4000 cm^−1^.

### 2.6. Nuclear Magnetic Resonance Spectroscopy (NMR)

The ^1^H NMR spectra were obtained based on the method previously described by Xie et al. [18]. Pectins were mixed with D_2_O and trisodium phosphate (TSP) (0.05%, *v*/*v*) at a concentration of 20 mg/mL at 298 K. ^1^H NMR spectroscopy was performed using a Bruker Avance III 600 MHz spectrometer (Bruker Technologies, Rheinstetten, Germany).

### 2.7. Rheological Properties of Different Pectins and Emulsion

Rheological tests were performed using an AR2000 Rheometer (TA Instruments, New Castle, DE, USA) as mentioned in the study by Huang, Zhao, Zhang, Liu, Hu and Pan [2]. Pectin (3% *w*/*v*) was dissolved into citric acid-sodium citrate buffer solution overnight. The pH of the solution was adjusted to 3.5, and the solution was placed between a cone-plate (40 mm diameter, 2° angle). The shear gap was set to 52 μm, and the selected measurement procedure was steady-state shear mode. The temperature was set to 25 °C with the shear rate ranging exponentially from 1 to 100 s^−1^. The rheological parameters including apparent viscosity and shear stress were obtained using the analysis software provided with the instrument.

### 2.8. Emulsifying Properties of Different Pectins

Pectin emulsion was prepared based on the procedure of Guo et al. [21]. Pectin (1% *w*/*v*) was dissolved into citric acid-sodium citrate buffer solution overnight. Sodium azide (0.05%) was added as a preservative, and the pH was adjusted to 3.5. Refined soybean oil (15 g, Arawana, Yihai Kerry group, Shanghai, China) was mixed into the pectin solution (20 g). The primary emulsion was emulsified for 3 min at a speed of 10,000 rpm using a high-speed homogenizer (Ningbo Xinzhi Biotechnology Co., Ltd., Ningbo, China). Thereafter, the solution was emulsified with a JY92-2D ultrasound processor (NingBo Scientz Biotechnology Co. Ltd., Ningbo, China) at 400 W for 10 min.

The droplet-size distributions of emulsions stored for 0, 7, 14, 21 and 30 days were determined by a computer-controlled laser diffraction apparatus (Mastersize 2000, Malvern Instruments Ltd., Worcestershire, UK), with the measurement ranging from 0.04 to 2000 μm. The volume average diameter D_4,3_ and surface average diameter D_3,2_ were recorded.

The microstructures of the emulsions stored for 0, 7, 14, 21 and 30 days were photographed with a 40× objective lens on an inverted microscope BIO 200-PH (Jingbaizhouxian technology Co. Ltd., Beijing, China). Microphotographs were taken using a Nikon D5000 digital camera (Nikon Corporation, Sendai, Japan) with the microscope and with at least 10 pictures taken per sample.

Emulsion stability was measured using both centrifugation and storage assessment [15]. Approximately 10 g of the pectin emulsion was centrifuged at room temperature for 15 min at 3000× *g*.

The ES_15_ was calculated with the following equation:

$${\mathrm{ES}}_{15}(\%)=\frac{\mathrm{Volume}\;\mathrm{of}\;\mathrm{emulsion}\;\mathrm{layer}}{\mathrm{Total}\;\mathrm{volume}\;\mathrm{of}\;\mathrm{fluid}}\;\times \;100$$

After storing the prepared emulsion at 4 °C for 7, 14, 21 and 30 days, the ES_storage_ was measured using the following equation:

$$\mathrm{ESstorage}\left(\%\right)=\frac{\mathrm{Remained}\;\mathrm{volume}\;\mathrm{of}\;\mathrm{emulsion}\;\mathrm{layer}}{\mathrm{Initial}\;\mathrm{volume}\;\mathrm{of}\;\mathrm{emulsion}\;\mathrm{layer}}\;\times \;100$$

### 2.9. Statistical Analysis

All experiments in this study were performed in triplicate, and the data were expressed as the means values ± standard deviation (SD). Analysis of variance (ANOVA) was conducted with SPSS 26. Statistical analysis was conducted with Tukey’s test, and the confidence level for statistical significance was set at a probability value of 0.05.

## 3. Results and Discussion

### 3.1. Extraction Yield of Pectin 

**Figure 1:**
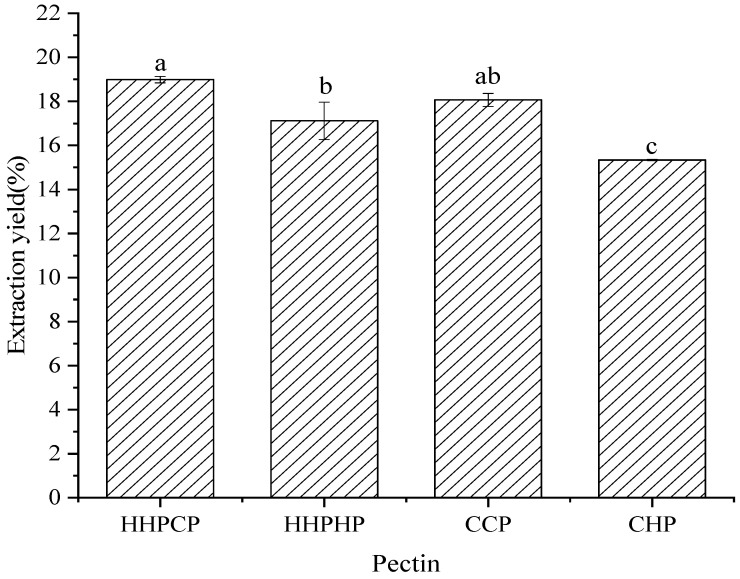
Effect of different extraction methods on extraction yields of Satsuma mandarin peel pectin. Note: different letters (a–c) denote significantly different in the row (*p* < 0.05).

The extraction yield of pectins from Satsuma mandarin peel using two different extraction techniques with two different solvents is shown in Figure 1, ranging from 15.34% to 18.99%. The pectin content in Satsuma mandarin peel obtained in this study was comparable to or higher than that reported in other citrus peels [8,19], indicating that Satsuma mandarin peel could be used as a potential pectin source.

As shown in Figure 1, when citric acid was used as the extractant, the extraction yields of pectin were significantly higher than those obtained with hydrochloric acid, both for HHPE and CE. This could be because some organic acids (such as citric acid, oxalic acid, etc.) had good chelation properties, which can significantly increase the solubility of insoluble pectin, yielding better extraction results. Similar results were also found, showing that citric acid extraction had a superior pectin yield compared to widely used sulfuric acid and hydrochloric acid [12], which resulted in a very branched pectin with an extremely high DE (83%) and a high *Mw* [13]. All of these results suggested that HHP-assisted citric acid extraction can be used as a promising approach with the characteristics of high efficiency and reduced time to extract pectin from Satsuma mandarin peel.

In addition, when hydrochloric acid was used as the extractant, the yields of HHPE were higher than those with CE. This might be because the cell wall of Satsuma mandarin peel was further damaged by HHP than with the conventional method, which could have enhanced the release of pectin and increased the extraction yield. These results were in agreement with previous studies on extraction of citrus by HHP, including pectin from lime peel [11] and pomelo peel [19]. Naghshineh, Olsen and Georgiou [11] reported that HHP (200 MPa/50 °C) resulted in significantly higher yields of pectin from lime peel than those obtained using aqueous extraction. Similarly, the yield with HHP (500 MPa/55 °C) was significantly higher than that from conventional heating methods, and the extraction time (10 min) was much shorter than that of conventional heating (60 min) [19].

### 3.2. Physicochemical Properties of Pectin

The nature and characteristics of pectin usually vary with different extraction conditions [13]. The GalA content of pectin obtained by different extraction methods is shown in Table 1. It could be observed that the GalA content of Satsuma mandarin peel pectin was 75.35–84.38%, which meets the requirements for commercial food grade pectin (GalA > 65%) [2]. Therefore, Satsuma mandarin peel pectin can be used as a promising source of commercial food-grade pectin. Moreover, there were no significant differences in GalA content for HHPCP, HHPHP, CCP and CHP, indicating that different extraction conditions had less noticeable effects on the GalA content. These results were consistent with a study on the effects of HHP (200 MPa/5 min) treatment on the GalA content of potato peel pectin [18]. Similarly, compared with apple pectin, there was no significant difference in the GalA content of the pectin after different pressure treatments (200, 400, 600 MPa), indicating that the main chain of pectin did not change [22].

The DE value is an important index for measuring the degree of methylation of galacturonic acid in pectin [23]. Pectin can be classified into high-methoxyl pectin (HMP, DE > 50%) and low-methoxyl pectin (LMP, DE < 50%) [24]. As shown in Table 1, all pectins appeared to be HMP, and no significant differences were observed in DE values for HHPCP, HHPHP, CCP and CHP (67.68–71.41%). The effect of HHP on DE values of pectin has already been reported; Guo, Zhao, Liao, Hu, Wu and Wang [10] obtained high-ester pectin from pomelo peel, but there were no significant differences in DE values of pectin extracted by HHP and high-shearing homogenization extraction. However, some published studies revealed that the DE value of pectin was related to different extraction solvents [25,26]. Citric acid-extracted pectin with microwave heating exhibited higher DE values than using hydrochloric acid [25]. The reason for the differences could be the extraction conditions, as strong acid may help to increase the de-esterification and fragmentation of polygalacturonic chains under the condition of microwave heating [26].

**Figure 2:**
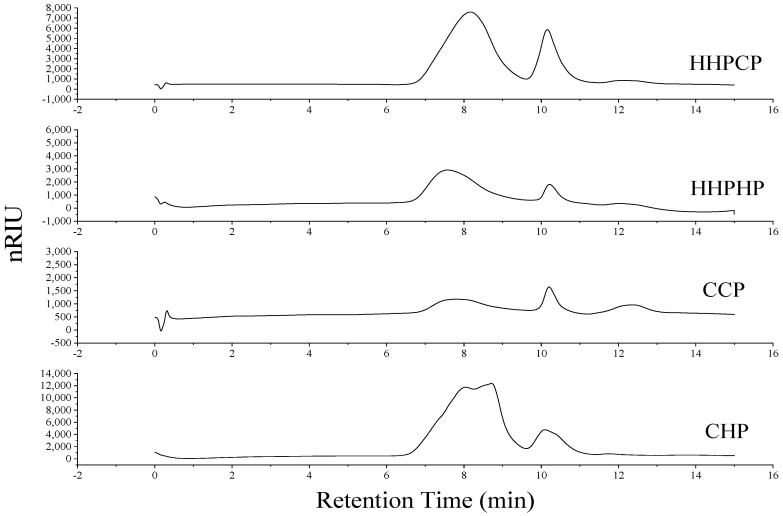
Effect of different extraction methods on molecular weight distribution of Satsuma mandarin peel pectin.

From the HPSEC results exhibited in Figure 2, there were significant differences in the molecular weight distribution of different pectins. Two peaks were observed for the molecular distribution for all pectin samples in this study. The *Mw* values of Satsuma mandarin peel pectin are very high (1201–2626 kDa), significantly higher than those of lime pectin (308–342 kDa) [11], pomelo pectin (437–520 kDa) [21], and navel orange peel (66.3–306.3 kDa) [19]. The higher *Mw* pectin extracted from Satsuma mandarin peel may exhibit good rheological and emulsifying properties [4,27]. Therefore, Satsuma mandarin peel can be used as a novel source of pectin.

In addition, the *Mw* value of HHPCP (1201 kDa) was significantly lower than that of HHPHP (2626 kDa) and the *Mw* value of CCP (1674 kDa) was lower than that of CHP (1871 kDa). These results indicate that citric acid as an extractant resulted in lower *Mw* values for pectin than hydrochloric acid, especially for HHP-assisted extraction. As discussed above regarding the yields of pectin, HHP could increase the yields of pectin compared to CE. It can be speculated that citric acid may help to chelate Satsuma peel pectin in some lower *Mw* ranges, while hydrochloric acid may help to extract pectin in higher *Mw* ranges, and these reactions may be accelerated by high-pressure processing. The effect of acid type on the *Mw* of pectin has already been reported. The *Mw* of sunflower pectin obtained by sodium citrate blending was about half the *Mw* of pectin extracted with oxalic acid-ammonium oxalic acid, suggesting that sodium citrate-extracted pectin underwent a deeper degradation in higher *Mw* ranges or that sodium citrate helped to extract pectin in lower *Mw* ranges [28]. Yang et al. [29] found that citric acid-extracted pectin from potato pulp exhibited the lowest *Mw* compared to those extracted with hydrochloric acid and sulfuric acid. Moreover, HHP may promote the dissolution of low molecular segment pectin or cause deeper degradation of high molecular pectin chains during the extraction process. Peng, Mu, Zhang, Sun, Chen and Yu [17] reported that as pressure increased (250–450 MPa), the *Mw* of beet pectin showed a downward trend. This could also partly help to explain the significantly lower *Mw* value for HHPCP. However, the effect of HHP-assisted acid extraction on the *Mw* of pectin may be complex and needs further investigation in following studies.

### 3.3. FTIR Spectra of Pectin

**Figure 3:**
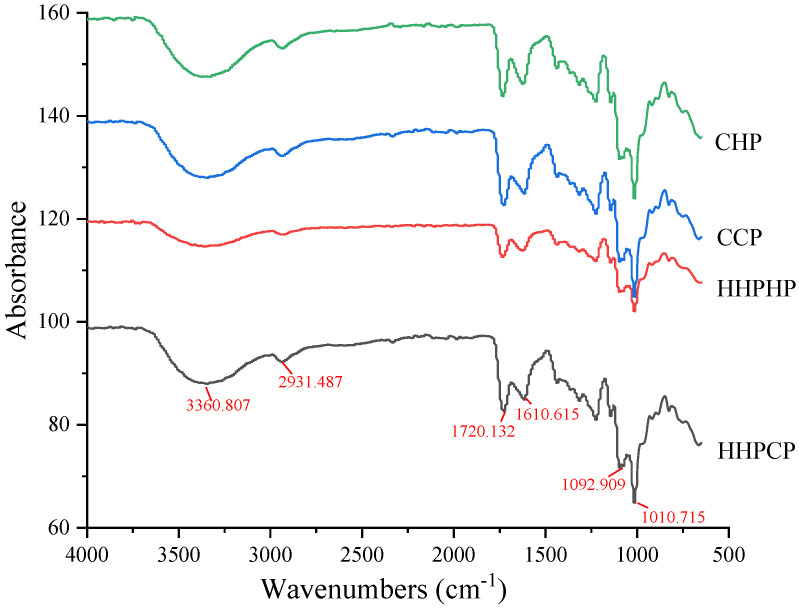
FTIR spectra of Satsuma mandarin peel pectin extracted by different extraction methods.

The FTIR spectra of pectin extracted by different methods are shown in Figure 3. The broad peaks appearing between 3600 and 3200 cm^−1^ were the result of the O-H stretching vibration, indicating the presence of intra-molecular and inter-molecular hydrogen bonds in the pectin molecule. The medium absorption peaks at approximately 2930 cm^−1^ were attributed to the C-H stretching of CH_2_ groups [18]. The absorption peaks at 1730 to 1760 cm^−1^ corresponded to the ester carbonyl (C=O) groups, and the strong absorption peaks at 1600–1630 cm^−1^ were caused by the vibration of COO^−^ in pectin carboxy groups, which were also the characteristic peaks of pectin different from neutral polysaccharide [15,30]. In this study, medium absorption peaks in the FTIR spectra of pectin at about 1720 cm^−1^ and 1610 cm^−1^ confirmed the presence of ester carbonyl (C=O) groups and carboxyl ion stretching band (COO^−^) in different pectins. All pectins obtained with different extraction methods were HMP, which was in agreement with the values obtained by titration as discussed above. The medium absorption peaks at about 1440 cm^−1^ were the characteristic peaks of the C-H variable angular vibration, and the peaks around 1220 cm^−1^ showed the C=O stretching of the O=C-O structure. The “fingerprint” area of a carbohydrate is the spectrum between the wavenumbers of 1200 to 800 cm^−1^ [31]. The study found that the samples had strong absorption at 1140, 1100 and 1020 cm^−^^1^, which were the characteristic absorption peaks of GalA in the fingerprint region of pectin polysaccharides. Similar bands were observed in pectin extracted from lime peel [25] pomelo peel [19] and Kara mandarin peel [5]. By comparing the changes in different pectins, all pectins showed a similar transmission mode, and the difference lay in the strength of each absorption peak. These results demonstrated that there were no significant differences in the group composition and relative content of pectin obtained by HHPE and CE.

### 3.4. ^1^H NMR of Pectin

**Figure 4:**
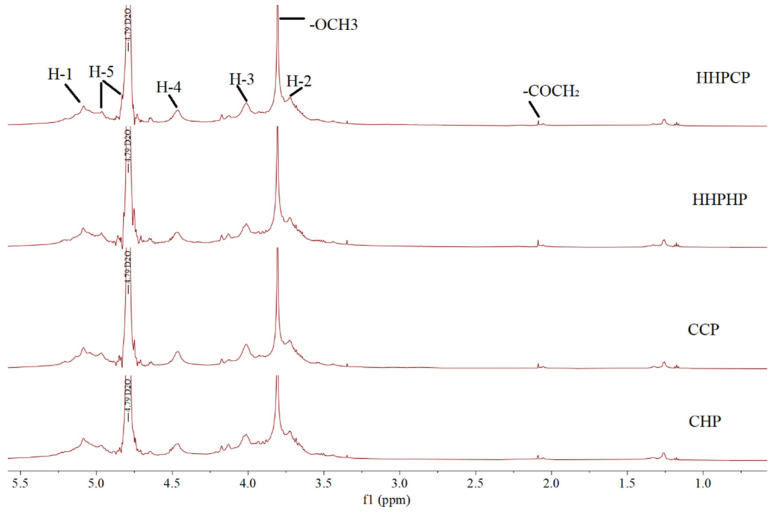
Effect of different extraction methods on ^1^H-NMR spectra of Satsuma mandarin peel pectin.

The ^1^H NMR spectra of different pectin are presented in Figure 4. The most significant peak located at 4.79 ppm was attributed to the solvent signal (D_2_O). The peak shown at δ 3.81 ppm could be attributed to the protons of the methoxy group of the galacturonic acid esterification unit [32]. The signals around 2 ppm were attributed to the acetyl esterified carboxyl groups of GalA units. The peaks that appeared around 1 ppm were probably due to the methyl group links of rhamnose [33]. The signals at about 5.1, 5.0, 4.5, 4.0 and 3.7 ppm were respectively related to the protons of H-1, H-5, H-4, H-3 and H-2 presented in the galacturonic acid units as discussed in other studies [34,35]. Additionally, the high intensity of chemical shifts of methoxy and acetyl groups (3.7 and 2.1, respectively) represented high DE values. It can be concluded that the pectins extracted by different methods showed higher DE values, confirming the data obtained from the titrimetric determination of DE. In view of the above discussion, the pectins extracted using different methods showed structural similarity.

### 3.5. Rheological Properties of Pectin

**Figure 5:**
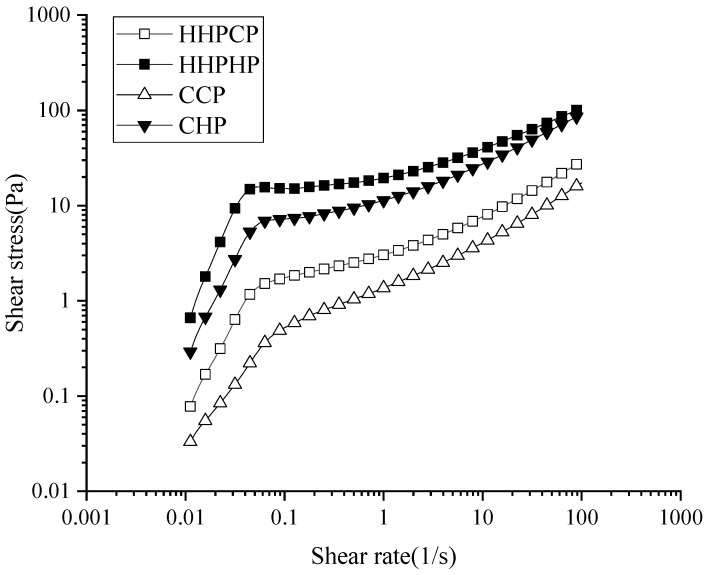
Rheological properties of solutions prepared from pectin extracted by different methods.

As shown in Figure 5, the shear stress of different pectin solutions increased with the increase in shear rate. Apparent viscosity refers to the ratio of shear stress to shear rate of a fluid during shearing. It can be deduced from the results that the apparent viscosity of the pectin solutions decreased with the increase in shear rate. All pectin solutions were non-Newtonian pseudoplastic fluids, exhibiting shear thinning behavior. Similar results for rheological characteristics were reported for lime peel pectin [25], sugar beet pectin [17] and mango peel pectin [2].

Apparent viscosity is often used as a primary criterion for assessing the thickening property and stability of pectin [15]. In this study, both for HHPE and CE, pectins using hydrochloric acid as extractant exhibited higher viscosity, which may be related to their higher *Mw*. This may be because macromolecules with higher molecular weights tend to disturb flow and thus exhibit higher viscosity. Wang, Huang, Fan, Zhao, Hu, Xu, Pan and Liu [4] reported that mango peel pectin extracted with the conventional method exhibited higher viscosity than those extracted with the ultrasound method, which was correlated to their higher *Mw.* Moreover, the apparent viscosity of pectin extracted by HHPE was higher than that of CE, regardless of the extraction solvents used in this study. Interestingly, the solution prepared from HHPCP had higher viscosity but with lower *Mw* values, as compared to the solution prepared from CCP. Except for *Mw*, the viscosity of pectin solutions could also be correlated to the DE value, GalA content and charge densities of pectin [1,18,36]. As discussed above, there were no significant differences in DE and GalA values for all pectins. Thus, the viscosity difference between HHPCP and CCP may be due to the charge densities. It has been reported that the pectin molecule with increased side chains could be obtained with HHP treatment, resulting in more charge exposure in the solution. The hydrophobic acetylated RG-I molecules in pectin, which may be clustered by hydrophobic associations through acetylated rhamnose residues, increased the viscosity to a much higher level [36]. Similarly, pectin treated with HHP (200 MPa) had a higher viscosity and better rheological properties than untreated pectin [18]. All of the above results demonstrated that different extraction methods had noticeable effects on the rheological characteristics of pectin and HHPHP could be a potentially better thickener.

### 3.6. Emulsifying Properties of Pectin

#### 3.6.1. Emulsion Viscosity

**Figure 6:**
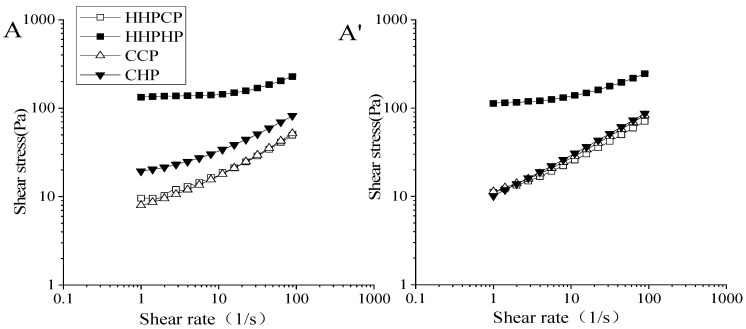
Flow curves of O/W emulsions prepared from pectin extracted by different methods at day 0 (**A**) and day 30 (**A’**).

Flow curves of O/W emulsions prepared from different pectins at day 0 and day 30 are exhibited in Figure 6. For the fresh emulsions, the viscosities of emulsions prepared from HHPCP and CCP showed no significant difference, while the viscosities of emulsions prepared from CHP were higher than those of HHPCP and CCP. After 30 days of storage, there were no significant differences in the viscosities of the emulsions prepared by HHPCP, CCP, and CHP. However, the viscosity of the emulsion prepared by HHPHP was significantly higher than that of the other three emulsions, both for the emulsions at day 0 and day 30. This phenomenon was consistent with the viscosity of the pectin solutions, indicating that the *Mw* values of pectin played an indispensable role in the viscosity of the emulsions. The higher the *Mw* values, the stronger the H-bond cross-linking effect of pectin, resulting in an increase in the viscosity and emulsion stability of the emulsion. Yang et al. [37] revealed that a higher *Mw* could allow pectin from pomegranate to possess a higher apparent viscosity when it was completely hydrated, thereby showing good ability to stabilize the emulsions. Citrus pectin with a higher *Mw* significantly increased emulsion stability [27]. In the present study, the emulsion prepared from HHPHP had the highest viscosity and *Mw*. The higher viscosity of emulsions can decrease the particle movement and coalescence effect and effectively increase emulsion stability [38].

#### 3.6.2. Emulsion Particle Size and Particle Size Distribution

**Figure 7:**
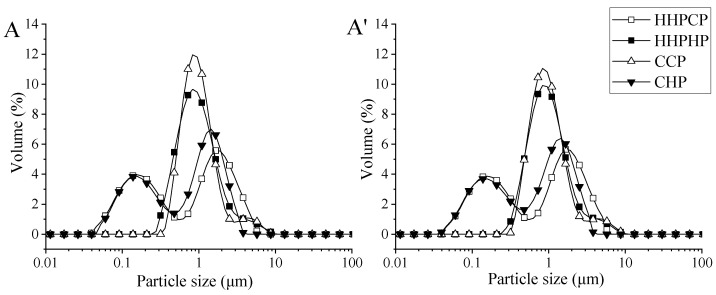
Particle size distribution of different pectin emulsions freshly prepared (**A**) and after storage (**A’**) at 4 °C.

The particle size distributions of different pectin emulsions at day 0 and day 30 are presented in Figure 7, and the average particle diameter (D_4,3_ and D_3,2_) of all emulsions is shown in Table 2. For fresh emulsions, those prepared with CCP and HHPHP exhibited a narrow and simple peaked particle size distribution, which showed larger particle size. HHPCP and CHP emulsions had a broader particle size distribution with two peaks and curves almost coincident. These findings were evidenced by the D_4,3_ and D_3,2_ values, with CCP and HHPHP emulsions showing larger values than emulsions prepared from HHPCP and CHP. The particle size distributions of pectin emulsions were related to *Mw*, DE value, GalA content and pectin structure. As discussed above, no significant changes were observed in DE and GalA values for all pectins. Therefore, the differences in the pectin emulsions could be attributed to *Mw* and pectin structure. The *Mw* values of Satsuma mandarin peel pectin (1201–2626 kDa) were higher than those of lime pectin (308–342 kDa) [11], pomelo pectin (437–520 kDa) [21], and navel orange pectin (66.3–306.3 kDa) [19]. The higher the *Mw* value, the stronger the cross-linking effect of pectin, resulting in an increase in stability of the emulsion. Wang, Huang, Fan, Zhao, Hu, Xu, Pan and Liu [4] found that as the *Mw* values increased, the particle size distribution curves of mango pectin shifted to the left, indicating that pectin with higher *Mw* values showed a smaller particle size and better emulsion stability. Additionally, the particle size distribution of pectin emulsions with different degrees of polymerization showed significant differences. Excessive depolymerization was not conducive to the stability of the emulsion, because too short polysaccharide chains could create a thin adsorption layer and thus could not achieve sufficient dimensional scale to stabilize the droplets [39]. However, the research on the influence of pectin structure on particle size distribution of pectin emulsions is still insufficient, and further investigations are needed.

During the 30 days of storage, the particle size of all emulsions was stable, and no obvious shift was observed in the particle size distributions of all emulsions, which indicated that pectin from Satsuma mandarin peel can form stable O/W emulsions. Generally, the particle size in emulsions tends to increase during the period of storage, mainly due to the movement and polymerization of the emulsion particles in emulsions [15]. However, in this study, the particle size in different pectin emulsions was stable during storage. The reason may be that the particle size in emulsions prepared from different pectins was 0.2 to 1.3 μm, which was significantly smaller than the particle size in citrus pectin emulsions in previous studies [39]. D_4,3_ values for the pomelo peel pectin emulsions with pH of 3–6 were between 8 and 31 μm [21], and D_4,3_ values for pectin from citrus peel were between 2 and 8 μm [39], which was higher than the D_4,3_ values in this study. In addition, in order to obtain a stable emulsion with small D_4,3_, a small molecular surfactant such as Tween or Span was usually used as an emulsifier, and a high-pressure method such as high-pressure homogenization was used [40]. However, Satsuma mandarin pectin emulsions prepared from different methods exhibited smaller average particle diameters that were close to those of nano-emulsions, and the process could be achieved easily. Based on the above results, it can be concluded that different extraction conditions have little effect on the particle size distribution of pectin and that Satsuma mandarin peel pectin can be a promising emulsion stabilizer thanks to its smaller particle size.

#### 3.6.3. Light Microscopy

**Figure 8:**
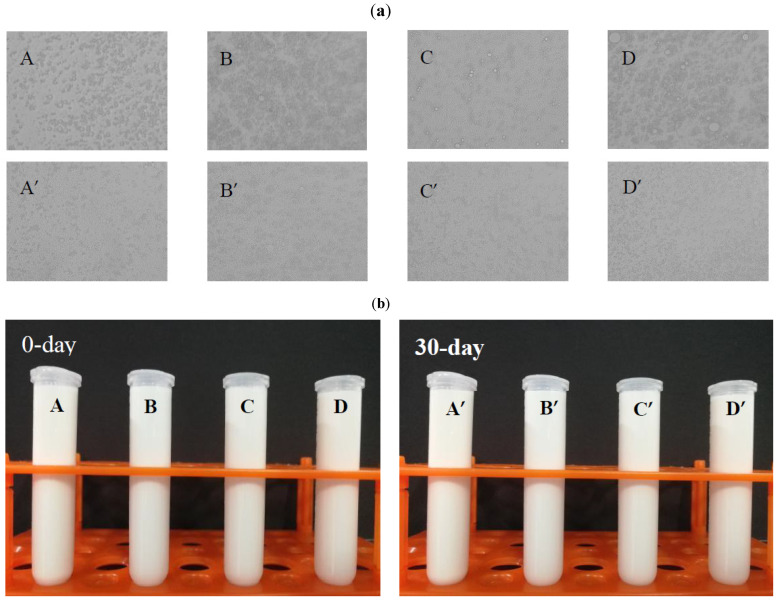
(**a**) Light micrographs of emulsions prepared by different extraction methods on day 0 and day 30 of storage (40×). (**b**) Stability of the O/W emulsions prepared by different extraction methods on day 0 and day 30 of storage. (A and A’: HHPCP; B and B’: HHPHP; C and C’: CCP; D and D’: CHP).

Since pectin emulsions are composed of numerous tiny globules, the size, shape, uniformity and composition of the granules in the emulsions can be observed by optical microscopy [41]. The microscopy images of the emulsions prepared from HHPCP, HHPHP, CCP and CHP are exhibited in Figure 8a. The results showed that the O/W emulsions prepared with different pectins consisted of regular spherical particles and had little difference in particle diameter and volume. Moreover, it could be observed that small droplets of different emulsions had some coalescence after storage for 30 days, but no significant increase occurred in the average particle diameter, which was consistent with the particle size distribution of emulsions. Usually, the particle diameter of the solution may increase during the storage process, which may contribute to the aggregation of pectin molecules and bridging flocculation between droplets. In the research by Wan, Chen, Huang, Liu and Pan [15], the coalescence of the emulsion prepared from pectin extracted with alkaline methods was obvious and manifested by a significant increase in the particle average diameter of the emulsion during storage. After storage of the citrus pectin and hawthorn pectin emulsion for 4 weeks, the particle size increased to some extent [42]. As discussed above regarding the particle size distributions, the results for pectin emulsions could be understood. These findings further confirmed the good emulsion stability of each pectin emulsion during storage.

#### 3.6.4. Emulsion Stability

The emulsion stability of different pectins was determined using a combination of centrifugation and storage assessment. The short-term emulsion stability assessed by centrifugation can reflect the ability of the droplets to resist re-agglomeration during the emulsification process. After 15 min centrifugation, the pectin emulsions prepared by different methods had the best ES_15_ (100%) and no evident phase separation was observed, which showed that all pectin emulsions exhibited good emulsifying stability in the centrifugation assay. These findings were consistent with the particle size distribution of emulsions. Figure 8b shows photographs of each pectin emulsion stored at 4 °C for 30 days. Compared with the centrifugation assay, the experimental results determined by the storage assessment could better reflect the change in the real emulsion under storage conditions. The results of the two methods were found to be consistent by comparison. After 30 days of storage, the ES_storage_ was still 100%, and all emulsions did not exhibit obvious delamination, showing the high stability levels of the emulsions from different pectins. As discussed above, it could be inferred that different extraction methods have little influence on the emulsion’s stability and pectin from Satsuma mandarin peel could produce and efficiently stabilize oil-in-water emulsions.

## 4. Conclusions

This study investigated the pectins extracted from Satsuma mandarin peel using high hydrostatic pressure or conventional methods in citric acid and hydrochloric acid. The results revealed that Satsuma mandarin peel could be used as a novel resource for pectin extraction with high emulsifying properties. No matter what methods and solvents were used, all pectin emulsions exhibited small particle average diameters, good centrifugation stability and long-term emulsion stability during storage. For different extraction conditions, citric acid as extractant could significantly increase the yield of pectin and result in lower *Mw* values of pectin than hydrochloric acid. HHPCP showed higher pectin extraction yield, while HHPHP exhibited higher *Mw*, resulting in higher viscosity and better rheological properties.

However, the mechanism of the effect of different extraction methods on the molecular structure of pectin needs to be further studied, and it is also necessary to deepen our understanding of the high emulsification mechanism of Satsuma mandarin pectin, so as to provide guidance for the preparation of potential pectin nano-emulsions.

## Figures and Tables

**Table 1:** Physiochemical properties of Satsuma mandarin peel pectin prepared from different methods.

Different Pectins	GalA Content (%)	DE Values (%)	*Mw* (kDa)
HHPCP	77.04 ± 3.06 ^a^	67.68 ± 1.57 ^a^	1201 ^d^
HHPHP	75.35 ± 0.23 ^a^	70.47 ± 0.20 ^a^	2626 ^a^
CCP	84.08 ± 3.97 ^a^	69.22 ± 1.20 ^a^	1674 ^c^
CHP	76.64 ± 1.14 ^a^	71.41 ± 0.59 ^a^	1871 ^b^

All results are expressed as mean ± SD, *n* = 3. Different letters (a–d) in the same column indicate a significant difference (*p* < 0.05). GalA: galacturonic acid, DE: degree of esterification, *Mw*: average molecular weight.

**Table 2:** Average particle size of O/W emulsions prepared from pectin extracted by different methods.

Different Pectins	Particle Mean Diameter (0-Day, μm)		Particle Mean Diameter (30-Day, μm)	
	D_3,2_	D_4,3_	D_3,2_	D_4,3_
HHPCP	0.247 ± 0.001 ^b^	1.113 ± 0.009 ^b^	0.260 ± 0.001 ^b^	1.128 ± 0.013 ^b^
HHPHP	0.781 ± 0.020 ^a^	1.157 ± 0.019 ^a^	0.805 ± 0.023 ^a^	1.200 ± 0.020 ^a^
CCP	0.840 ± 0.005 ^a^	1.156 ± 0.029 ^a^	0.839 ± 0.011 ^a^	1.273 ± 0.013 ^a^
CHP	0.264 ± 0.001 ^b^	0.881 ± 0.001 ^c^	0.273 ± 0.001 ^b^	0.888 ± 0.000 ^c^

All results are expressed as mean ± SD, *n* = 3. Different letters (a–c) in the same column indicate a significant difference (*p <* 0.05).

## Data Availability

Not applicable.

## Sample Availability

Samples of the compounds are available from the authors.
